# Recent Applications of Liposomes in Ophthalmic Drug Delivery

**DOI:** 10.1155/2011/863734

**Published:** 2011-03-01

**Authors:** Gyan P. Mishra, Mahuya Bagui, Viral Tamboli, Ashim K. Mitra

**Affiliations:** Division of Pharmaceutical Sciences, School of Pharmacy, University of Missouri-Kansas City, 2464 Charlotte Street, Kansas City, MO 64108-2718, USA

## Abstract

Liposomal formulations were significantly explored over the last decade for the ophthalmic drug delivery applications. These formulations are mainly composed of phosphatidylcholine (PC) and other constituents such as cholesterol and lipid-conjugated hydrophilic polymers. Liposomes are biodegradable and biocompatible in nature. Current approaches for topical delivery of liposomes are focused on improving the corneal adhesion and permeation by incorporating various bioadhesive and penetration enhancing polymers. In the case of posterior segment disorders improvement in intravitreal half life and targeted drug delivery to the retina is achieved by liposomes. In this paper we have attempted to summarize the applications of liposomes in the field of ophthalmic drug delivery by citing numerous investigators over the last decade.

## 1. Introduction

Ocular drug delivery is challenging in terms of achieving optimum drug concentration due to unique protective mechanisms of the eye. Development of a drug delivery system for attaining therapeutic concentration at the target site requires a comprehensive understanding of static and dynamic barriers of the eye. The eye has two broadly defined segments, (a) anterior segment, and (b) posterior segment. Anterior segment is the front one-third of the eye that includes the optical structure in front of vitreous humor: cornea, pupil, aqueous humor, iris, lens and ciliary body. Posterior segment is the back two-thirds of the eye that mainly includes sclera, choroid, retina, vitreous humor, macula, and optical nerve. The common routes of drug administration for the treatment of eye disorders are topical, systemic, periocular, and intravitreal. Topical administration is the most preferred route because of highest patient compliance and least invasive nature. Upon topical instillation, absorption of drugs takes place either through corneal route (cornea, aqueous humor, intraocular tissues) or noncorneal route (conjunctiva, sclera, choroid/RPE). The cornea can be mainly divided into the epithelium, stroma and endothelium, where each layer offers a different polarity and a potential rate-limiting structure for drug permeation. The non-corneal route involves absorption across the sclera and conjunctiva into the intraocular tissues. However, a small fraction of the topically applied drugs, generally less than 5%, reaches the intraocular tissues. Factors responsible for poor ocular bioavailability following topical instillation are precorneal drainage and lipoidal nature of the corneal epithelium. In addition, a major fraction of drug reaches the systemic circulation through conjunctival vessels and nasolacrimal duct, which leads to severe adverse effects. Consequently, topical route has met with limited success in attaining therapeutic drug concentrations in the posterior segment. Systemic administration can provide therapeutic levels in the posterior segment, but administration of high doses is necessary, which often leads to severe side effects. Blood-aqueous barrier and blood-retinal barrier are the two major barriers for anterior segment and posterior segment ocular drug delivery, respectively, after systemic administration. The tight junctional complexes located in the two discrete cell layers, the endothelium of the iris/ciliary blood vessels, and the nonpigmented ciliary epithelium offer blood-aqueous barrier which prevents the entry of solutes into the aqueous humor. Blood retinal barrier is composed of two types of cells, that is, retinal capillary endothelial cells and retinal pigment epithelium (RPE) cells which prevents the entry of solute into the retina. Intravitreal administration requires frequent administration which may lead to high susceptibility for vitreous hemorrhage, retinal detachment and endophthalmitis. These side effects can be minimized by developing delivery systems which provide controlled and targeted drug delivery for prolonged periods [1–3]. Conventional ophthalmic formulations such as solutions and suspensions exhibit poor bioavailability. Over the last decade, numerous drug delivery systems have been explored to overcome the limitation of conventional dosage forms. Novel formulations such as nanoparticles, liposomes, dendrimers, and niosomes were developed to enhance drug bioavailability and to minimize adverse effects [4, 5]. Among them liposomal formulations were widely explored in the last decade for drug delivery applications.

**Figure 1:**
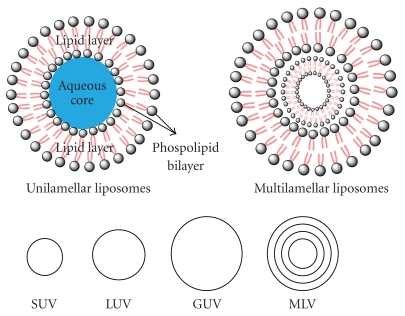
Schematic representation of basic structures and different types of liposomes.

In 1965 liposomes were first introduced as the drug delivery carriers [6]. Liposomes are usually within the size range of 10 nm to 1 *μ*m or greater. These vesicular systems are composed of aqueous core enclosed by phospholipid bilayers of natural or synthetic origin. Liposomes are structurally classified on the basis of lipid bilayers such as small unilamellar vesicles (SUVs) or multilamellar vesicles (MLVs). Furthermore, on the basis of size, liposomes are classified into small unilamellar vesicles (SUVs), giant unilamellar vesicles (GUVs), and large unilamellar vesicles (LUVs) (Figure 1). Unilamellar vesicles are composed of single layer of lipid such as lecithin or phosphatidylglycerol encapsulating aqueous interior core. Multilamellar vesicle is composed of various layers of lipid bilayers [7–9]. MLVs are metastable energy configuration having different facets depending upon the polydispersity of the liposomal formulation. Various types of liposomes with size are summarized in Table 1.

Drug loading capacity of liposomes depends on many factors such as size of liposomes, types of lipid utilized for preparation, and physicochemical properties of therapeutic agent itself. For example, being the smallest in size entrapping efficiency for SUVs is poor in comparison to MLVs. However, LUVs provide a balance between size and drug loading capacity. Liposomes are advantageous in encapsulating both lipophilic and hydrophilic molecules. Hydrophilic drugs are entrapped in the aqueous layer, while hydrophobic drugs are stuck in the lipid bilayers. Loading capacity of ionic molecules can be further improved by using cationic or anionic lipids for the preparation of liposomes [11].

Majority of liposomal formulations utilize phosphatidylcholine (PC) and other constituents such as cholesterol and lipid-conjugated hydrophilic polymers as the main ingredients. Incorporation of cholesterol enhances the stability by improving the rigidity of the membrane. Stability of liposomes depends upon the various properties such as surface charge, size, surface hydration, and fluidity of lipid bilayers. Surface charge determines interaction of liposomes with ocular membrane. Positively charged liposomes display better corneal permeation than the neutral and negatively charged liposomes. Neutral liposomes upon systemic administration evade the elimination by reticuloendothelial system (RES). However, these vesicles possess higher self-aggregation tendency. In contrast, negatively and positively charged liposomes exhibit lower aggregation tendency but undergo rapid clearance by RES cells due to higher interaction with serum proteins. In addition, size of the liposomes can also regulate the clearance by RES. Liposomes of size less than 100 nm generally exhibit significantly higher circulation time due to decrease in opsonization of liposomes with serum protein [13]. 

Amphiphilic nature of phospholipids allows these molecules to form lipid bilayers. This unique feature is utilized for the preparation of liposomes. In general, hydration of phospholipids results in the formation of MLVs, which can be processed into SUVs with proper sonication. However, addition of aqueous solution of surfactant above the critical micelle concentration results in the formation of phospholipids micelles. After the dialysis of surfactant aggregation of micelles form LUVs, critical micelle concentrations of amphiphiles which can form micelles are four to five orders of magnitude higher than the phospholipids which form liposomes [12]. Numerous methods have been reported to prepare liposomes. Most commonly solvent evaporation method, reverse phase evaporation method and detergent dialysis method are employed [14]. The encapsulated drug from liposome can be released either through passive diffusion, vesicle erosion, or vesicle retention. In passive diffusion, drug molecules tend to penetrate through the lipid layers of liposome to reach extra vesicular layer either by diffusion or convection mechanism. The rate of diffusion depends on the size, lipid composition, and the properties of the drug itself [15–17]. Unilamellar liposomes exhibit faster release rate than multilamellar ones because in multilayered liposome, drug diffusion occurs through a series of barriers; hence, the drug release is delayed. Phospholipase and high-density lipoprotein present in blood plasma can damage phospholipid layers of liposome and thus results in vesicle erosion and releases the encapsulated drug into the cell. The drug release rate depends on the extent of liposomal membrane damage [18]. Liposome-cell interactions depend on several factors like size, surface charge, composition of liposomes, targeting ligand on the surface of liposome, and biological environment. Liposomes can interact with cells by four different mechanisms: adsorption, fusion, lipid exchange and endocytosis (receptor mediated). Liposomes can be specifically or nonspecifically adsorbed onto the cell surface or can be fused with cell membranes, and release encapsulated drug inside the cell. During adsorption, liposomes can release encapsulated drug in front of cell membrane, and released drug can enter cell via micropinocytosis. They can also be engulfed inside the cell by specific or nonspecific endocytosis process. Negatively charged liposomes have been found to be more efficient than neutral liposomes for internalization into the cells by endocytosis process. Liposomes bind to the receptor present in the invaginations of cellular membrane and are internalized into the cell by endocytotic pathway. After endocytosis, they can fuse with the endosomal membrane to form endosome which can be delivered to lysosomes. In lysosomes, the presence of peptidase and hydrolase degrades the liposomes and their content. To avoid this degradation and thus to increase cytoplasmic bioavailability, stimuli-responsive liposomes (such as pH or temperature) have been developed. pH-sensitive liposomes can undergo fusion with endosomal membrane and release their content directly into cytosol. In some cases liposomes become destabilized inside the endosome and release their content, or they destabilize endosomal membrane resulting in leakage of encapsulated content into cytosol [19, 20]. In this paper we have attempted to summarize the application of liposomes in the field of ophthalmic drug delivery attempted by numerous investigators over the last decade.

## 2. Application of Liposomes in Ophthalmic Drug Delivery

Liposomes have been investigated for ophthalmic drug delivery since it offers advantages as a carrier system. It is a biodegradable and biocompatible nanocarrier. It can enhance the permeation of poorly absorbed drug molecules by binding to the corneal surface and improving residence time. It can encapsulate both the hydrophilic and hydrophobic drug molecules. In addition, liposomes can improve pharmacokinetic profile, enhance therapeutic effect, and reduce toxicity associated with higher dose. Owing to their versatile nature, liposomes have been widely investigated for the treatment of both anterior and posterior segment eye disorders. Current approaches for the anterior segment drug delivery are focused on improving corneal adhesion and permeation by incorporating various bioadhesive and penetration enhancing polymers. However, in the case of posterior segment disorders, improvement of intravitreal half-life and targeted drug delivery to the retina is necessary. Currently verteporfin is being used clinically in photodynamic therapy for the treatment of subfoveal choroidal neovascularization (CNV), ocular histoplasmosis, or pathological myopia effectively. Verteporfin is a light-activated drug which is administered by intravenous infusion. In photodynamic therapy, after the drug is injected, a low-energy laser is applied to the retina through the contact lens in order to activate verteporfin that results in closure of the abnormal blood vessels. Unfortunately, photodynamic therapy usually does not permanently close the abnormal vessels and choroidal neovessels reappear after several months. Another liposomal photosensitizing agent, rostaporfin, was evaluated for the treatment of age-related macular degeneration. It is now under phase 3 clinical trial. Rostaporfin requires less frequent administration compared to verteporfin. Liposome technology has been explored for ophthalmic drug delivery. However, there are some issues to be addressed such as formulation, and storage of liposomes is very difficult, and they are known to cause long-term side effects. Intravitreal administration of liposomes has resulted in vitreal condensation, vitreal bodies in the lower part of eye, and retinal abnormalities. Therefore, all these factors should be taken into account while developing liposomal formulation for ophthalmic application [21–25]. Recent applications of liposomal formulations encapsulating various therapeutic molecules are summarized in Table 2.

## 3. Topical Applications

In 1981, Samolin et al. investigated the role of liposomes in ophthalmic drug delivery. Since then several investigators proposed strategies to enhance absorption of drugs having poor physicochemical properties. Studies performed by Schaeffer and Krohn suggested the role of charge and size in transcorneal permeation. Investigators observed four-fold higher *in vitro* corneal flux from penicillin G-loaded SUVs. They reported corneal permeation in the order of SUV+  >  MLV− >  SUV−  >  SUV  > MLV free drug. These studies explored the role of vesicle type on transcorneal permeation across the excised rabbit cornea [36]. Role of physicochemical property of entrapped drug was elucidated by other investigators. Liposomal formulation of TA produced twofold increase in drug concentration in both the cornea and aqueous humor in the rabbit model. On the contrary, liposomal formulation of hydrophilic drug, that is, dihydrostreptomycin sulfate, did not improve the corneal permeation [37, 38]. Considering these findings, it was evident that both vesicle type and physicochemical property of drug significantly affects the transcorneal flux of the formulation. 

Earlier investigation by Fitzgerald et al. was significant in exploring the clearance of liposomes by gamma scintigraphy following topical administration in the rabbit model. These investigators reported, SUVs with positive charge had improved the corneal retention by interacting with negatively charged corneal surface. Since then, approaches based on positively charged liposomes were explored considerably. Researchers also explored immunoliposomes, lectin functionalized liposomes, and positively charged lipid analogs. Among these approaches only immunoliposomes did not improved liposome-corneal interaction. However, lectin and lipid analog-based approaches are not explored considerably in the field of ophthalmic drug delivery [39].

**Figure 2:**
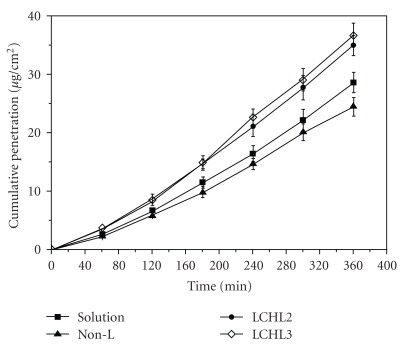
Corneal penetration profiles of diclofenac sodium in different vehicles, noncoated liposome (Non-L), LCHL2 (0.25% Low molecular weight chitosan, w/v), and LCHL3 (0.5% low molecular weight chitosan, w/v), (with permission from [10])

**Figure 3:**
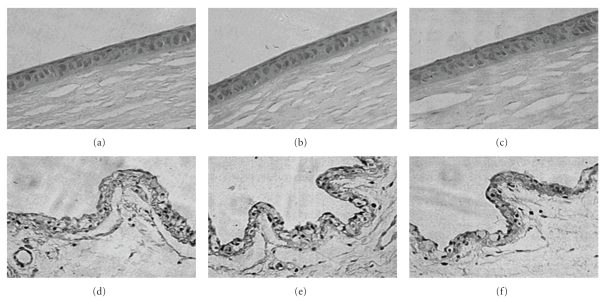
Histopathology microscopy of the ocular tissues after being treated with LCHL for 7 days. cornea of nontreated (a), treated with LCHL2 (b), and LCHL3 (c); conjunctiva of nontreated (d), treated with LCHL2 (e) and LCHL3 (f) (with permission from [10]).

Approach of utilizing chitosan in the formulation was reported to be advantageous in improving the precorneal residence time due to its mucoadhesive nature. Degradation of chitosan into oligosaccharides is mediated through lysozymes, and degradation products are nontoxic in nature [40, 41]. Biodegradable nature is advantageous for selecting chitosan in the formulation of ocular drug delivery systems. Topical administration of chitosan-coated liposomes (chitosomes) improves precorneal retention and also slows down drug metabolism at the precorneal epithelial surface. Chitosan-based mucoadhesive liposomal formulation of CPX was prepared and evaluated by Mehanna et al. Reverse phase evaporation technique was utilized for the preparation of liposomes, which were further coated with chitosan of different molecular weights. The authors reported that liposomes coated with high molecular weight chitosan were smaller in size due to complete coverage of liposomal surface, which acted as a physical barrier to inhibit aggregation. In addition, authors determined lower encapsulation efficiency (EE) of 49.93% for coated liposomes in comparison to uncoated negative and neutral liposomes with 71.4% and 53.2% EE, respectively, due to electrostatic repulsion between chitosan and cationic drug. The effect of liposomal surface charge on the particle size was also determined. Negatively charged liposomes were larger in diameter due to predominantly electrostatic attraction between the positively charged chitosan and negatively charged phospholipids. Rheological studies revealed ideal pseudoelastic behavior of chitosomes and higher apparent viscosity than the liposome dispersion. The author suggested that pseudoelastic property of chitosome provides prolonged retention and stability of tear film. Moreover, *in vitro * release studies with chitosomes exhibited slower drug release rate in comparison to free liposomes due to additional diffusion barrier for drug molecule. *Ex vivo * corneal permeation studies across isolated rabbit cornea suggested that due to absorption enhancing nature of chitosan relative permeability of chitosomes was 1.74-fold higher than free drug. Furthermore, *in vitro* antibacterial studies revealed that chitosomes exhibited enhanced antibacterial activity than the marketed aqueous solution against reference and clinically isolated strains of *Pseudomonas aeruginosa *and* Staphylococcus aureus. *Authors suggested the electrostatic interaction of positively charged chitosan and negatively charged bacterial cell wall enhanced the antibacterial action of liposomal formulation. Comparative single dose *in vivo* study performed on bacterial conjunctivitis rabbit model revealed that chitosomes inhibited the growth of *Pseudomonas aeruginosa *for 24 h. It was reported that marketed product (Clioxan) is comparatively less effective and requires frequent administration. These investigators demonstrated the role of medium molecular weight chitosan. However, other studies suggest the advantages of water-soluble low molecular weight chitosan as potential biopolymer for coating liposomes [42]. Application of LCH was advantageous in eliminating the aggregation behavior of chitosan at physiological pH that had dramatically influenced *in vivo* performance of the liposomal formulation. Investigator reported higher *ex vivo* corneal penetration across excised rabbit cornea in the case of LCH-coated liposomes as shown in Figure 2. However, higher concentration of LCH (0.25% and 0.5% w/w) did not show significant change in particle size. Researchers suggested that a loose coating layer is responsible for aggregation of vesicles which resulted in higher particle size in the case of 0.1% w/v LCH. Moreover, the drug release at 6 h was 38.9% in noncoated liposomes whereas only 25.4% drug release was observed in liposomes coated with 0.25% w/v chitosan solution. Both nontreated and treated group did not demonstrate any abnormality of the corneal and conjunctival epithelial cells. In addition, no ocular irritation and inflammatory response was observed. *In vitro *precorneal retention studies in rabbits showed that the elimination of chitosan-coated liposomes was slower than non-coated liposomes. Authors suggested that mucin film, which primarily covers the surface of cornea and conjunctiva, is composed of negatively charged glycoprotein. Electrostatic alteration between positively charged LCH and mucin promotes prolonged retention. In addition, hydrogen bonding interactions of LCH with the ocular surface also favors precorneal retention. This study demonstrated the role of LCH in improving the precorneal retention. However, previous studies with high molecular weight chitosan-coated liposomes did not improve the precorneal retention due to enhanced intramolecular interactions. Histopathological analysis of the LCH-coated liposomes in rabbits after long-term irritation test revealed that the formulation was biocompatible with the ocular tissues (Figure 3) [10].

Application of quaternized derivatives of chitosan that is, N-trimethyl chitosan chloride (TMC), with significantly higher water solubility at physiological pH, was evaluated for surface modification of coenzyme Q10-loaded liposomes. Improved stability of the modified liposomes was reported. In addition, surface modification with cationic polymeric film reduced particle aggregation through stearic stabilization and improved precorneal retention than uncoated liposomes due to ionic interaction with negatively charged corneal surface. Investigators reported almost 4.8-fold increase in precorneal residence time measured by gamma scintigraphy after administration of 25 *μ*L of formulation. Histological analysis and draize test performed on rabbits revealed that TMC was biocompatible with corneal epithelium. Moreover, higher molecular weight TMC exhibited better anticataract activity in Sprague Dawley rats [43]. To take the dual advantage of chitosan-based nanoparticles and liposomes, Diebold et al. prepared liposome-chitosan nanoparticle complexes [44]. As mentioned earlier, chitosan nanocarriers were employed in topical drug delivery because of its mucoadhesive nature, whereas liposomes can incorporate variety of drug molecules and improve ocular drug bioavailability [45–47]. These nanosystems were formulated as eye drops, which possessed combined properties of both carriers and overcome the ocular mucosal barriers. These authors evaluated the nanosystems for toxicity on spontaneously immortalized epithelial cell line from normal human conjunctiva (IOBA-NHC). Cells pre incubated with XTT (2,3-bis[2-methoxy-4-nitro-5-sulfophenyl]-2H-tetrazolium-5-carboxyalinide) solution (1 mg/mL XTT in 100 mL of phenol red-free RPMI culture medium) were exposed to different concentrations of chitosan nanoparticles and liposome-chitosan nanoparticles complexes. Cytotoxicity was determined by measuring the production of yellow color due to cleavage of XTT by mitochondrial enzymes. Cell viability after exposure of liposome-chitosan nanoparticle complexes was higher in comparison to chitosan nanoparticles alone. They also performed *in vivo* acute tolerance test by administrating the formulations topically to the female albino New Zealand rabbits. The nanosystems did not show any evidence of toxicity to the both sham-controlled and treated eyes. No sign of irritation on ocular surface was confirmed by clinical microscopic sign score. Also, *in vivo* experiments have shown that nanosystems can enter the conjunctival cells without causing histological alteration to the cornea, conjunctiva, and lid tissues in the rabbit model. In addition, the complexes did not release any inflammatory mediators in cornea, conjunctiva, and eyelids [44].

Vaccination approach can successfully overcome the limitations of antiviral agents in the treatment of HSV infections. However, delivery of vaccines is the major hurdle facing by pharmaceutical scientists. Administration by conventional parenteral route has several drawbacks such as high cost, need of highly trained personnel, and needle-stick injuries. Cationic liposomes containing herpes simplex virus (HSV) antigens were proposed as potential carriers, in the form of a periocular vaccine, to protect animals against subsequent HSV-1 ocular challenges. Two different peptides, namely, DTK1 and DTK2 (DTKs), having antiherpetic activity were synthesized. Cationic liposomes containing both DTK and secretory HSV-1 glycoprotein B were formulated. Liposomal formulation showed effective results in a rabbit model of HSV-1 infection [29].

Zhang et al. utilized cytochrome-C (Cyt-C) loaded cationic liposomes for the treatment of selenite-induced cataract in rats. These liposomes were fabricated by thin-layer evaporation technique. Authors investigated the effect of composition on the encapsulation efficiency. This study reported improvement in the entrapment efficiency (EE) with increasing phosphatidylcholine component, whereas EE was lowered by incorporating stearylamine. Cyt-C loaded freeze-dried liposomes were stable for one year at 4°C. Furthermore, these liposomes exhibited remarkable efficacy (28% decrease in lens opacity) in minimizing the cataract formation. Liposomal encapsulation of Cyt-C has significance, but the preparation method adapted by these authors was similar to previous investigations [48].

In another study, fluconazole liposomal formulation was evaluated in the candidal keratitis model in rabbits. In this investigation, comparative efficacy of the fluconazole solution and fluconazole-loaded liposomes was determined. The purpose of developing liposomal formulation was to prolong the antifungal action by increasing the contact time. In the rabbits treated with fluconazole solution, 50% healing was observed in 3 weeks, whereas 86.4% healing was observed in rabbits treated with fluconazole encapsulated liposomes. Authors attributed enhanced pharmacological activity to higher viscosity and lipid solubility of fluconazole-loaded liposomes [49].

Chronic ocular infectious diseases such as conjunctivitis, bacterial keratitis need high drug concentration at the site of infection. Treatment of these diseases requires frequent eye drop administrations that may cause drug resistance and also decrease patient compliance. In order to minimize precorneal drainage and increase bioavailability viscosity enhancers such as poly (vinyl alcohol) and polymethacrylic acid were blended with eye drop solution [50]. Many investigators evaluated the role of liposomal hydrogel formulation for the delivery of fluoroquinolone antibiotics. For example, liposomal hydrogel formulation of ciprofloxacin (CPX) was reported to avoid tear-driven dilution in the cul de sac. Lecithin (LEC) and *α*-L-dipalmitoylphosphatidylcholine (DPPC) were utilized as major ingredients in the preparation of multilamellar liposomes. Poly (vinyl alcohol) (PVA) and polymethacrylic acid (PMA) derivatives were utilized for gel formulation. Various formulation parameters such as viscosity and rheological property of liposomes was evaluated in relation to the *in vitro* release [51]. CPX because of its negative charge electrostatically interacts with lipid head group of the phospholipid bilayers [52]. Therefore, majority of drug was entrapped inside the liposomes. Similar electrostatic interaction between lipid bilayers and other fluoroquinolones such as ofloxacin and lomefloxacin were reported in other studies [53]. The investigator observed that use of viscosity enhancing agents in the formulation had affected the drug release rate. The addition of gel forming agents PVA and PMA did not affect the rigidity of liposomal membrane, instead these polymers were adsorbed on the surface of multilamellar liposomal surface because of method of formulation. Hydration of lipids with proper concentration of PVA and PMA results in the formation of polymer layer on the surface of the liposomes [54, 55]. Direct correlation was observed between viscosity of hydrogel and drug release rate. In addition, they found a remarkable difference in drug release half-time between two different lipids, that is, LEC and DPPC. The presence of unsaturated lipid in LEC provides less rigid structure to the liposome formulation that resulted in faster drug release in comparison to DPPC. Hydrogel formulation has shown plastic properties; that is, under higher shear stress condition, it remained in free flowing state, whereas it exhibited no flow state at rest. Overall, the use of optimized formulation of liposomal hydrogel can sustain the release of antibacterial agents in comparison to liposomes alone, and this approach could be beneficial in the treatment of various chronic ocular infectious diseases. In another study, CPX-loaded liposomal hydrogel formulation improved transcorneal permeation in rabbit model. Liposomes were suspended in the hydrogel matrix composed of carbopol 940. The investigators reported that drug entrapment efficiency was enhanced with the increase of cholesterol concentrations, which provided higher stability and lower permeability of lipid bilayers. Furthermore, higher encapsulation efficiency with positively charged liposomes was observed due to favorable electrostatic attraction between CPX and cationic stearylamine. Liposomes of higher size were obtained upon incorporation of charge inducing agents, which expand lipid bilayers distance. Positively charged liposomes exhibited slower release rate, and CPX release was more sustained from the liposomes suspended in the carbopol gel because of additional barriers for diffusion. Liposomal hydrogel displayed fivefold higher *in vitro* transcorneal permeation across excised rabbit cornea than the aqueous solution. This approach was already explored by other investigators. Although authors observed enhanced *in vitro* transcorneal permeation, it would be interesting to evaluate these formulations for *in vivo* studies, where tear dilution plays a major role [27]. In a similar study by these researchers, transcorneal permeation of ofloxacin-loaded thermosensitive liposomal hydrogel was evaluated. Two different types of liposomes, MLV and reverse phase evaporation vesicles (REV), were prepared. Authors observed smaller particle size with REV relative to MLV due to differences in the method of preparation. Splicing of the lipid monolayer in a more curved structure resulted in REV of smaller diameter. Authors evaluated chitosan/*β* glycerophosphate thermosensitive hydrogel system. Incorporation of liposomes in thermosensitive gels reduced the gelling time from 5 to 1 minute. The researchers suggested that hydrophobic interaction can reduce energy requirement for gelation. Transcorneal permeation studies across excised rabbit cornea revealed sevenfold higher drug permeation from the liposomal formulation than ofloxacin aqueous solution. This effect was observed due to mucoadhesive nature of the hydrogel base which prolonged the retention of formulation across the excised rabbit cornea. In addition, cationic nature of chitosan in the thermogelling system promoted corneal adherence and opened corneal epithelial tight junctions. Researchers concluded that ofloxacin liposomal formulation will reduce the formation of crystalline deposit and also frequency of administration. Another investigation suggests threefold increase in corneal residence of ophthalmic formulation containing chitosan. The ocular irritation test suggests excellent tolerance of chitosan formulation evaluated with confocal laser scanning ophthalmoscope [56, 57]. A liposomal spray formulation was recently evaluated for changes in preocular tear film. After application of the spray, liposomes traverse to the tear film. Liposomal formulation was evaluated on human subjects, and effectiveness was compared to normal saline at different time points. Authors reported statistically significant improvement in tear film stability and lipid layer stability in comparison to control. These studies suggest the potential of liposomal sprays in the treatment of dry eye syndrome [58].

Liposomes were also investigated for the topical delivery of intraocular pressure (IOP) lowering agents. For example, acetazolamide was encapsulated in liposomes to enhance the solubility and corneal permeation. Liposomes were formulated by reversed phase evaporation and liquid hydration methods with and without the use of positive or negative charge inducers to prepare REV and MLV. Liposomes of different compositions were evaluated for entrapment efficiency, stability, *in vitro* release, and IOP lowering efficacy in rabbit model. The entrapment efficiency of acetazolamide was found highest with positively charged liposomes followed by neutral and negatively charged liposomes because of ionic interaction between anionic drug and lipid bilayers. Cationic and neutral MLVs of acetazolamide exhibited maximum effectiveness in terms of release profile for the same reason [30]. Another IOP reducing agent, demeclocycline (DEM), was encapsulated in liposomes which enhanced ocular permeability. This formulation achieved long-lasting IOP lowering effects relative to pilocarpine liquid formulation [59]. Monem et al. reported pilocarpine HCl loaded liposomes which were administered topically. This study reported two different liposomes with neutral and negatively charged multilamellar surface. Neutral liposomes were more effective in IOP lowering effect than negatively charged liposomes or free drug. In addition, phase transition and size distribution studies showed long term stability (15 months) of the liposomal formulation [60]. 

**Figure 4:**
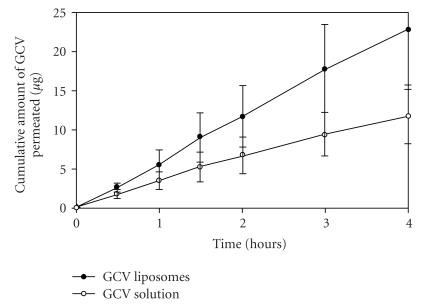
In vitro transcorneal permeation of GCV liposomes and solution (with permission from [26]).

**Figure 5:**
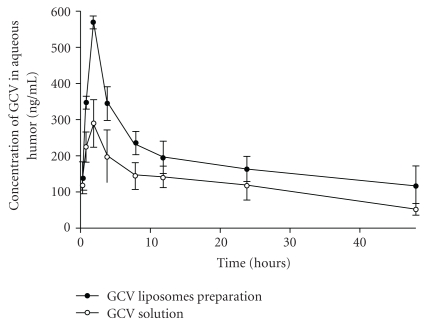
Concentration-time profile of GCV in aqueous humor after instillation of 1 mg/mL GCV liposome preparation and GCV solution in rabbits (with permission from [26]).

Liposomal formulations were also developed for the delivery of antiviral agents. Shen and Tu reported the application of liposomes for the delivery of ganciclovir (GCV) to the vitreous humor via topical administration in the rabbits. GCV liposomes were prepared by the reversed phase evaporation method utilizing PC/CH/sodium deoxycholate mixture. *In vitro* transcorneal permeability and *in vivo* ocular pharmacokinetics of the liposomal formulation were compared with the GCV solution. Transcorneal permeability was 3.9-fold higher (Figure 4), and ocular bioavailability of GCV liposomes was 1.7-fold greater in comparison to solution (Figure 5). GCV concentrations from liposomal formulation were 2 to 10 times higher in various ocular tissues. In addition, *in vivo* experiments suggested that the scleral pathway contributed in the absorption of GCV liposomes, as the highest concentration of GCV was obtained in the sclera. Concentrations of GCV attained in the cornea and the sclera were higher than IC_50_ value of GCV against CMV. The author suggested that the particle size (i.e., 200 nm) and composition of the liposomes played a major role in transocular permeation [26]. 

Disposable contact lenses presoaked with medication solution have been utilized for continuous drug delivery. However, in presoaked contact lenses, drug molecules randomly disperse within the contact lenses and show burst release that can cause local tissue toxicity or other side effects [61]. To avoid rapid drug release and to provide site-specific delivery, another novel strategy, liposomes loaded soft contact lenses, was proposed for the antibiotics in the treatment of ocular infections such as bacterial keratitis. Multilamellar liposomal surface of soft contact lenses was decorated with PEG-Biotin linkage. Contact lenses with surface-immobilized levofloxacin-loaded liposomes followed first-order release kinetics and released the drug over more than 6 days. In addition, the liposomal formulation has shown antibacterial activity against *S. aureus* [28, 62]. In another study, chloramphenicol (CAP) was encapsulated in dimyristoylphosphatidylcholine (DMPC) liposomes and formulated in the form of eye drops. Three methods, that is, CAP-PART (partitioning of CAP in the vesicle bilayers), CAP-EN (entrapment of CAP via normal hydration method), and CAP-ADS (adsorption of CAP on the vesicle surface) were employed for the preparation of liposomes. The formulation was evaluated for interaction of the drug with the phospholipid bilayers resulting in optimum efficacy against *S. aureus*. CAP was localized in the interfacial lipid bilayers in the case of CAP-EN whereas entrapped deeper in the bilayers in the case of CAP-PART. These results showed that CAP located near the interfacial region within the hydrophobic core of the liposomes had shown highest anti-bacterial activity against *S. aureus* for almost 5 hrs [63]. 

Chetoni et al. reported acyclovir (ACV) containing positively charged unilamellar liposomes (LIPO-ACV), administered topically into rabbit eyes. The bioavailability of LIPO-ACV was compared with free ACV in solution (SOL), ACV encapsulated in “empty” liposomes (LIPO-EMPTY), and a diluted dose of commercially available ACV ointment, containing same ACV concentrations (0.12%). The pharmacokinetic profile of the drug in the aqueous humor of rabbits showed highest drug concentration profile for LIPO-ACV system with 90 minutes plateau. LIPO-ACV exhibited aqueous humor ACV concentration in the upper range of the ID_50_ (0.01 to 0.7 *μ*g/mL). In a separate study concentrated ACV ointment (containing 8-fold greater dose of ACV) was compared with LIPO-ACV. Only 1.6 times higher bioavailability was observed with ACV ointment. These results indicate a significant advantage of LIPO-ACV as an alternative to ACV ointment [47]. In order to give an insight on release mechanism of ACV from liposomal vehicle, *in vitro *release experiments through a cellophane membrane was performed which showed lower drug release from the liposomal vehicle through cellophane membrane compared to that of SOL and LIPO-EMPTY. These results sustained the concept that negatively charged corneal epithelium enhances the efficacy of positively charged liposomal formulation.

Pleyer et al. formulated different cationic liposomes by changing their lipid compositions in order to improve gene expressions in corneal endothelial cells. The authors reported six formulations with different cationic lipids 3*β*[N-(N,N′-dimethylaminoethane)-carbamoyl] (DAC), dicarbobenzoxyspermin-carbamoyl (SP), N-Amidino-*β*-alanin-[2-(1,3-dioleoyloxy)propyl]amid-hydrochlorid (DOSGA), and 1,2-dimyristyloxypropyl-3-methylhydroxethylammoniumbromide (DMRIE) which were coupled in varying concentrations with neutral lipid dioleoylphosphatidylethanolamine (DOPE). Fixed amount of DNA was entrapped in each liposome which expressed *E. coli * beta-galactosidase. Transfection experiments on bovine corneal endothelial cells (BCEC) indicated that SP20 (SP/DOPE 20/80) generated highest efficiency followed by DMRIE 50 (DMRIE/DOPE 50/50) ranging at approximately 3 mU per *β*-gal per well. The researchers observed low gene expressions with DAC 30 (DAC/DOPE 30/70), and DOSGA 30 (DOSGA/DOPE 30/70), DOSGA 100 (DOSGA 100) and no gene expressions for free DNA. At a fixed DNA concentration, the relative *β*-galactosidase expressions were decreased with increasing the cationic lipid dose, which might be due to either toxic effects of cationic lipids at higher concentrations to the cells or non-optimal lipid/DNA ratios. The highest efficiency of SP20 liposomes in delivering DNA into BCEC can be rationalized by considering its rapid and stable complexation with DNA due to result of ionic interactions between the multivalent lipid and negatively charged phosphate groups of DNA. SP20 was completely biodegradable compared to many synthesized lipids as it was derived from naturally occurring compounds resulting in least toxicity compared to other liposomal formulations [64]. 

Teshima et al. studied prednisolone- (PLS-) incorporated liposomes to improve retention property of prednisolone. Introduction of a lipophilic moiety (palmitoyl) to prednisolone (Pal-PLS) greatly enhanced drug retention in liposomes as lipophilic moiety increased its affinity to liposomal lipid bilayer. The investigators studied two liposomes containing two different lipids, egg phosphatidylcholine (EggPC) and distearoyl phosphatidylcholine (DSPC). Ultrafiltration and gel filtration techniques were used to investigate retention properties of PLS and pal-PLS in liposomes. While ultrafiltration method showed high incorporation efficiency of PLS into the liposomes, a significant decrease of its incorporation efficiency was observed in gel filtration. This result indicated that elution medium in gel filtration studies released incorporated PLS from liposomes. Pal-PLS showed higher incorporation efficiency in both ultrafiltration and gel filtration studies. However, incubation of liposomes with rat plasma for 1 min effectively decreased Pal-PLS incorporation into EggPC/Chol liposomes as detected by gel filtration. The reducing effect of Pal-PLS incorporation into liposomes by rat-plasma was overcome by using DSPC lipid in liposomal formulation. Further surface modification of liposomes with a hydrophilic polymer PEG resulted in the protection of the entrapped palmitoyl-PLS and thus generated a stable retention property of the drug [65].

Law et al. reported topical administration of acyclovir- (ACV)- encapsulated liposomes, where *in vitro* corneal penetration and in vivo corneal absorption (using male rabbits) of acyclovir from ACV-encapsulated liposomes were studied. This study reported the effect of liposomal surface charge on their corneal penetration and absorption. Surface charge of liposomes plays a significant role in improving the efficiency of ocular drug delivery system. Positively charged liposomes exhibited higher drug loading efficiencies as well as faster drug release rates compared to negatively charged liposomes. Prolonged penetration across the cornea was observed for ACV-encapsulated liposomes. This phenomenon was more evident in case of positively charged liposomes. The penetration rate for positively charged liposomes was found to be approximately 3.6-fold lower than free ACV and approximately 2-fold lower than negatively charged liposomes. Similarly, ACV concentration profile in aqueous humor indicated higher corneal absorption and greater corneal deposition of ACV for positively charged liposomes relative to negatively charged ACV and free ACV. The researchers suggested that positively charged liposomes can interact electrostatically with the negatively charged surface of cornea. This interaction can result in stronger binding which leads to formation of a completely coated layer on the corneal surface. This layer may cause an increase in residence time on the cornea surface resulting in higher ACV absorption and greater extent of ACV deposition in the cornea compared to that of negatively charged liposomes [46]. 

Kawakami et al. reported O-palmitoyl prodrug of tilisolol-encapsulated liposome to improve the retention time of tilisolol in the precorneal area and vitreous body. The liposomes were administered topically, as well as intravitreally to the rabbit eye. Following topical administration, the researchers observed very low retention of O-palmitoyl tilisolol in the tear fluid even when it was applied as liposomal formulation. The investigators significantly increased the retention property of liposomes by adding 2% of carmellose sodium which acted as a reservoir for liposomes. In case of intravitreal administration, o-palmitoyl tilisolol-encapsulated liposomes responded well resulting in higher drug concentration in the vitreous body compared to free tilisolol [66]. 

In the last decade numerous researchers addressed the challenge of minimizing rapid clearance from precorneal site and enhancing the corneal permeation through various approaches. Utilization of chitosan in the preparation of mucoadhesive and cationic formulations was widely explored for the delivery of small therapeutic molecules from different categories. Other mucoadhesive polymers were also applied in the formulation of hydrogels that can regulate the drug release rate at the ocular surface.

## 4. Intravitreal Applications

Liposomes represent the first injectable systems for intravitreal administrations. Liposomes can provide sustained release for prolonged period. In addition, liposomal formulation can minimize the tissue toxicity and enhance the intravitreal half-life of drugs by decreasing rapid clearance from vitreous cavity [67, 68]. 

Barza et al. delineated the effect of liposome size and pathological state of eye on the intravitreal elimination kinetics of carriers. Investigators observed that the clearance rate of SUVs was faster than LUVs. Moreover, intraocular inflammation also increases the intravitreal clearance rate [69]. Recently ocular pharmacologists have utilized liposomal hydrogel and sterically (pegylated) stabilized liposomes to address the drawbacks associated with intravitreal administrations of liposomes [70].

In an application, rhodamine-conjugated liposomes loaded with vasoactive intestinal peptide (VIP) were given intravenously to healthy rats to examine efficacy in the treatment of ocular inflammation. VIP is an immunomodulatory neuropeptide involved in the regulation of ocular immune response by modulating the activities of macrophages, T lymphocytes, and dendritic cells [19, 71]. Intravitreal application of VIP-loaded liposomes was proposed for the treatment of endotoxin-induced uveitis [72]. Internalization of rhodamine-conjugated liposomes (Rh-Lip) alone and loaded with VIP (VIP-Rh-Lip) was examined in male Lewis rats. Intraocular and systemic biodistributions of the liposomes were also determined. The authors reported that, after single intravitreal injection, liposomes were internalized by retinal Müller glial cells, resident macrophages, and rare infiltrating activated macrophages. Majority of the liposomes reached the cervical lymph nodes via conjunctival lymphatics. VIP-Rh-Lip internalized via macrophages resulted in slower release and long-term expression inside the ocular tissues and cervical lymph nodes. Thus, intravenous delivery of VIP by liposomes would be helpful in the treatment of uveitis and other immune-mediated eye diseases by modulating the immune microenvironment of the ocular region [32].

Camelo et al. evaluated the liposomal formulation dispersed in hyaluronic acid (HA) gel for the delivery of VIP in the treatment of uveitis and uveoretinitis in Lewis rats. Major limitation with the VIP-LP was shorter residence time in the vitreous cavity due to rapid elimination through the lymphatic circulation. Investigators attempted to increase the half-life of VIP-loaded liposomes (VIP-LP) after intravitreal administration by suspending them in the hydrogel. HA which is the major component of vitreous was utilized for the studies. The researchers incorporated liposomes in HA gel in order to attain sustained release of VIP from the liposomes. VIP-LP suspended in HA gel was retained in the vitreous cavity for 8 days after single intravitreal injection. Authors reported that incorporation of liposomes in the gel had increased the viscosity of the gel due to the enhanced interaction between HA gel and phospholipids. Moreover, it was reported that formulation was effective in the treatment as evident by reduced clinical score and number of polymorphonuclear cells [73]. 

In a study tacrolimus-loaded liposomes were utilized for the treatment of uveoretinitis. The vesicles were prepared by reverse phase evaporation technique and subsequently evaluated for efficacy and safety following intravitreal injection in rats. No change in the retinal function was observed in the liposome-treated rats. Histopathological examination revealed reduced inflammatory response in comparison to free drug. Liposomes were able to maintain the vitreous concentration of more than 50 ng/mL for 2 weeks after single administration. Investigators concluded that tacrolimus-loaded liposomes were more effective than the drug alone. The formulation also reduced drug-related toxicity to inner retinal cells [31]. 

In another study, Abrishami et al. prepared nanoliposomes of bevacizumab. The investigators utilized dehydration-rehydration method for achieving highest encapsulation efficiency. Researchers attempted to reduce the clearance of bevacizumab liposomes by incorporation of cholesterol. In comparison to free drug, concentration of liposomal formulation was 5 times higher at 42 days. This study revealed that liposomal formulation of bevacizumab was proven effective in the controlled release of bevacizumab for more than 6 weeks in rabbit model [74]. 

Fluconazole liposomes were evaluated for the treatment of candidal endophthalmitis. In the comparative study, intravitreal injections of fluconazole solution or liposomal formulation were given at different dose levels in the rabbit eyes. Administration of fluconazole solution caused photoreceptor disorientation and ultrastructural changes of the retina at the concentration of 100 *μ*g in 0.1 mL or above. In contrast, liposomal formulation of fluconazole did not show any retinal alteration up to concentration of 200 *μ*g in 0.1 mL [75].

Cheng et al. Formulated lipid prodrug of ganciclovir (GCV), 1-O-hexadecylpropanediol-3-phospho-GCV into liposomes which were injected intravitreally in rabbits. The researchers used this liposomal formulation for antiviral treatment against herpes simplex virus type 1 (HSV-1) and human cytomegalovirus (HCMV). Intravitreal injection with 0.2 nM intravitreal concentration was the most effective without causing any side effects of vitreous clarity or cataracts development in the eye. Moreover, this formulation provided complete retinal protection even after simultaneous intravitreal injection [76].

Bochot et al. reported that phosphodiester oligonucleotide encapsulated sterically (pegylated) stabilized liposomes which were administered intravitreally in rabbits. It was the first reported use of liposomes as vehicle for intravitreal delivery of phosphodiester oligonucleotides. The investigators tried to overcome the problem of short intravitreal half-life of oligonucleotide by encapsulating [33P] labeled 16-mer oligothymidylate (PdT16) within liposome. After intravitreal injection liposomal formulations yielded significantly higher concentration of radiolabeled 33P within the posterior segment of the eye (vitreous, retina, choroid, and sclera) than the solution. A heterogeneous competitive hybridization assay revealed a significantly improved intraocular stability of PdT16 when it was administered in a liposomal formulation. The sterically stabilized hydrophilic polyethylene glycol (PEG) chains on the liposome's surface protected them from degradation, resulting in prolonged residence time in vitreous and sustained release of encapsulated oligonucleotide into the vitreous and the retina-choroid. Controlled release of [33P] PdT16 from liposomes also inhibited unwanted distribution of oligonucleotide in the nontargeted tissues (sclera, lens) and thus reduced overall ocular toxicity [77].

Peeters et al. reported cationic liposomes as nonviral gene carriers which were complexed with therapeutic DNA, called lipoplexes (LPXs). The authors investigated the factors responsible for inefficient vitreous diffusion of nonviral gene complexes and addressed the problems to overcome vitreous barrier for lipoplexes. FITC-dextran, fluorescent polystyrene nanospheres as models for LPXs and LPXs were mixed with vitreous gel obtained from bovine eyes, and their mobility in vitreous was studied by fluorescence recovery after photobleaching (FRAP) technique. Polystyrene nanospheres can bind to collagen fibers within the vitreous due to hydrophobic interactions resulting in restricted mobility in the vitreous. To overcome this problem, hydrophilic polyethylene glycol (PEG) chains were grafted on the surface of nanoparticles that had prevented adsorption to the collagen fibers and thus increased their mobility in the vitreous. They reported that the size of the nanospheres should be less than 500 nm to obtain good vitreous mobility; otherwise it would be sterically hindered by vitreous network and spread nonhomogeneously throughout the vitreous resulting in accumulation near the injection site. Nonpegylated cationic liposomes aggregated in the vitreous as negatively charged glycosaminoglycans (GAGs) strongly bind to the cationic lipoplexes, which neutralize positive zeta potential of lipoplexes, and thus favor aggregation. Low to moderate pegylation (1.9 mol% DSPE-PEG to 9.1 mol% DSPE-PEG) on cationic lipoplexes prevented their aggregation but, binding to biopolymers in the vitreous still occurred. Further increase of DSPE-PEG to 16.7 mol% prevented both vitreous aggregation as well as binding to vitreous fibrils, resulting in homogeneous vitreous distribution and vitreous mobility [35]. The size and zeta potential of pegylated LPXs decreased with increasing the amount of pegylated lipids (DSPE-PEG) in LPXs. Gel electrophoresis experiments indicated that LPXs in vitreous remain stable and do not disassemble. The data on mobility, aggregation, and stability of lipoplexes opened up a new direction to nonviral ocular gene therapy, but some factors need to take into consideration. Here transport of drugs in vitreous was assumed by diffusion mechanism only but in case of larger animal species like humans drug transport through convection plays a significant role. Moreover here transport was focused in the central parts of vitreous samples. Cortical vitreous zone containing densely packed collagen and inner limiting lamina may produce additional barriers to the diffusion of LPXs into the retina.

Gupta et al. evaluated fluconazole-encapsulated liposomes which were administered intravitreally in rabbit eyes. Entrapping of fluconazole into liposomes significantly slowed down clearance of free fluconazole after intravitreal injection and thereby achieved higher fluconazole concentration in the vitreous. The liposomes showed longer half-life (23.40 h) in comparison to free fluconazole (3.08 h) [78]. Among all these investigations performed by numerous researchers, approach of entrapping bevacizumab will be advantageous for designing controlled release system for therapeutic macromolecules. Another approach of using sterically stabilized liposomes for oligonucleotide delivery can be further explored for resolving the challenges in ocular gene therapy. This approach will be advantageous in minimizing the intravitreal clearance of liposomes and distribution of oligonucleotide in the non-targeted tissues.

## 5. Subconjunctival Applications

Subconjunctival mode of administration has gained new momentum in delivering the drugs to both the anterior and posterior segments [79]. Subconjunctival injection of liposomes can provide retentive effect and steady-state release at the site of application. Therefore, higher drug concentrations can be achieved at the target site. In addition, subconjunctival injection is better in comparison to topical application as it can improve patience compliance by avoiding repeated administrations and provide direct access of the drug to the target site [80, 81]. Absorption rate of liposome-bound low molecular weight heparin (LMWH) was investigated after subconjunctival injection in the treatment of subconjunctival hemorrhage (SH) in rabbits. Low concentration of liposome-bound LMWH was observed as compared to the free LMWH in the intraocular regions (aqueous and vitreous). Moreover, lower systemic level of LMWH was noted after subconjunctival injection. The paper suggested that, due to larger size (approx. 550 nm in size), liposomes remained at the site of injection and avoided lymphatic drainage. Also, positively charged liposomes encapsulated higher amounts of LMWH and released the drug in a sustained manner, thus providing longer residence time and increased concentration at the targeted site. Thus, subconjunctival application of liposomes is a possible strategy to avoid systemic side effects of LMWH [81]. In a similar study, Baek et al. attempted subconjunctival administration of streptokinase- (SK-) loaded liposomes for the treatment of SH in rabbits. Freeze thaw method was utilized for the production of liposomes. The study reported that 81% of the drug was released in 48 h. Higher absorption efficiency of liposomes in comparison to free drug was observed. SK-encapsulated liposomes in the early phase of SH need to be assessed [82].

Fukushima et al. reported clodronate liposomes (CL_2_MDP-lip), which were used to inhibit infiltration of macrophages in the conjunctiva in the case of blepharo conjunctivitis (EC) developed in Brown Norway rats. The liposomes were administered by subconjunctival injection as well as by intravenous injection. They found that CL_2_MDP-lip effectively decreased the number of ED2-positive macrophages in the conjunctivas, where ED1-positive macrophages infiltration could only be controlled if the injection was administered just prior to OVA challenge [33]. Limited investigations on subconjunctival delivery of liposomes were performed in the last decade. However, approach of utilizing liposomes of size greater than 550 nm can be explored in future for long-term delivery by minimizing the systemic clearance of liposomes through conjunctival capillaries. It would be interesting to investigate the subconjunctival clearance of liposomes of various sizes.

## 6. Conclusion

Numerous applications of liposomes in ophthalmic drug delivery were extensively studied. These carriers have successfully improved the drug bioavailability by controlled and targeted delivery. In the case of topical application improvement in the precorneal retention, transcorneal permeation, and therapeutic efficacy was achieved by utilizing liposomal formulations. In addition, effects of charge and composition of liposomes were explored in detail, which have provided comprehensive understanding of the interaction between liposome and ocular tissues. The applications of chitosan and hydrogel for improving the precorneal retention of liposomes were explored and shown potential for further investigation. Liposomal formulations have been evaluated for encapsulation of various drug molecules of different therapeutic classes. In particular, liposomal formulation of small molecules for the treatment of bacterial conjunctivitis and glaucoma was developed. Moreover, posterior segment delivery of liposomes was proven successful in enhancing the intravitreal half-life and targeted delivery to the inner retinal cells. In the case of posterior segment disorders liposomal formulation of therapeutic macromolecules was examined. However, research on targeted delivery of liposomes was limited. Receptors expressed on the cornea and retina could be explored in future for targeted drug delivery utilizing surface-modified liposomes.

## Figures and Tables

**Table 1:** Size of different types of liposomes [12].

Vesicle type	Size
SUVs	~20 nm to ~200 nm
LUVs	~200 nm to ~1 *μ*m
MLVs	*>*0.5 *μ*m
GUVs	*>*1 *μ*m

**Table 2:** Application of liposomes for the delivery of various drug molecules.

Drug	Formulation	Result	Year	Ref
GCV	Liposomes	*In vitro * transcorneal permeation and *in vivo * ocular pharmacokinetics was improved	2007	[26]
Ciprofloxacin	Liposomal hydrogel	Fivefold higher transcorneal permeation than the liposomes alone	2010	[27]
Levofloxacin	Liposomes attached to the contact lens	Drug was released following first-order kinetics for more than 6 days and formulation had showed activity against *S. aureus. *	2007	[28]
Herpes simplex virus antigens	Periocular vaccine	Treated rabbits showed anti-gB immune response and protected against reactivation of HSV infection	2006	[29]
Acetazolamide	Neutral- and surface-charged liposomes	Positively charged liposomes reduced IOP and exhibited prolonged effect than negatively charged liposomes	2007	[30]
Tacrolimus	Liposomes	More than 50 ng/mL vitreous concentration was maintained for 2 weeks and reduced drug related toxicity	2010	[31]
Vasoactive intestinal peptide	Rhodamine-conjugated liposomes	Liposomes were internalized by retinal Müller glial cells, resident macrophages; majority of the liposomes reached the cervical lymph nodes and resulted in slower release and long-term expression inside the eye	2007	[32]
Clodronate	Liposomes	Effectively inhibit infiltration of ED2-positive macrophages	2005	[33]
Plasmid DNA	Cationic liposomes	Significantly increased transfection efficiency of pDNA	2004	[34]
Therapeutic DNA	Cationic lipoplexes	Achieved good vitreous mobility with moderately pegylated cationic lipoplexes with size less than 500 nm	2005	[35]
